# Effectiveness of beinaglutide in a patient with late dumping syndrome after gastrectomy: A case report

**DOI:** 10.1097/MD.0000000000026086

**Published:** 2021-05-28

**Authors:** Bo Ding, Yun Hu, Lu Yuan, Reng-Na Yan, Jian-Hua Ma

**Affiliations:** Department of Endocrinology, Nanjing First Hospital, Nanjing Medical University, Jiangsu, China.

**Keywords:** continuous glucose monitoring, dumping syndrome, glucagon-like peptide-1 receptor agonists

## Abstract

**Rationale:**

Dumping syndrome is a frequent and potentially severe complication after gastric surgery. Beinaglutide, a recombinant human glucagon-like peptide-1 (GLP-1) which shares 100% homology with human GLP-1(7-36), has never been reported in the treatment of dumping syndrome before.

**Patient concerns:**

The patient had undergone distal gastrectomy for gastric signet ring cell carcinoma 16 months ago. He presented with symptoms of paroxysmal palpitation, sweating, and dizziness for 4 months.

**Diagnosis:**

He was diagnosed with late dumping syndrome.

**Interventions and outcomes:**

The patient was treated with dietary changes and acarbose for 4 months before admitted to our hospital. The treatment with dietary changes and acarbose did not prevent postprandial hyperinsulinemia and hypoglycemia according to the 75 g oral glucose tolerance test (OGTT) and continuous glucose monitoring (CGM) on admission.

Therefore, the patient was treated with beinaglutide 0.1 mg before breakfast and lunch instead of acarbose. After the treatment of beinaglutide for 1 month, OGTT showed a reduction in postprandial hyperinsulinemia compared with before starting treatment, and the time in the range of 3.9 to 10 mmol/L became 100% in CGM. No side effect was observed in this patient during beinaglutide treatment.

**Lessons:**

These findings suggest that beinaglutide may be effective for treating post-gastrectomy late dumping syndrome.

## 1 Introduction

Dumping syndrome is a frequent and potentially severe complication after gastric surgery.^[1]^ Late dumping syndrome is characterized by a rapid influx of carbohydrates into the upper jejunum, rapidly increased absorption into the intestine, insulin hypersecretion, and clinical hypoglycemia 2 to 3 hours after a meal.

Although glucagon-like peptide-1 receptor agonists (GLP-1RAs) are reported as a treatment of glucose lowering by the inhibition of glucagon secretion and promotion of insulin secretion in patients with type 2 diabetes,^[2]^ and one of the underlying causes of late dumping syndrome is thought to be the postoperatively elevated glucagon-like peptide-1 (GLP-1) levels lead to pancreatic beta cell hypertrophy,^[3,4]^ liraglutide, a GLP-1RA shares 97% homology with human GLP-1, has recently been reported as useful for late dumping syndrome.^[5–7]^ Exenatide, another GLP-1RA, shares less homology with human GLP-1 than liraglutide, was also used in one case of dumping syndrome.^[7]^ Beinaglutide, a new recombinant human GLP-1, shares 100% homology with human GLP-1(7-36). In this clinical case, we describe the successful use of beinaglutide in a patient with late dumping syndrome after gastrectomy who failed with acarbose treatment.

## 2 Case presentation and management

A 53-year-old man, who 16 months previously had undergone distal gastrectomy for gastric signet ring cell carcinoma (stage Ia), presented with symptoms of paroxysmal palpitation, sweating, and dizziness since February 2020. These symptoms could be remitted by eating. He was diagnosed with dumping syndrome due to a blood glucose level of 1.8 mmol/L 2 hours after breakfast without islet cell tumor in April 2020. Since then, he has been treated with acarbose (25 mg, three times a day), and dietary changes (5–6 meals per day with more protein and fiber, and less carbohydrates). However, these treatments failed. In the recent 1 month, he had 4 episodes of hypoglycemia (with blood glucose 1.8–3.0 mmol/L), most of which occurred 2 to 3 hours after breakfast, and was admitted to our hospital on August 26, 2020. In addition, he was also diagnosed with anxiety in April and was taking Escitalopram Oxalate 10 mg once a day. There was nothing to note in his family history.

**Figure 1:**
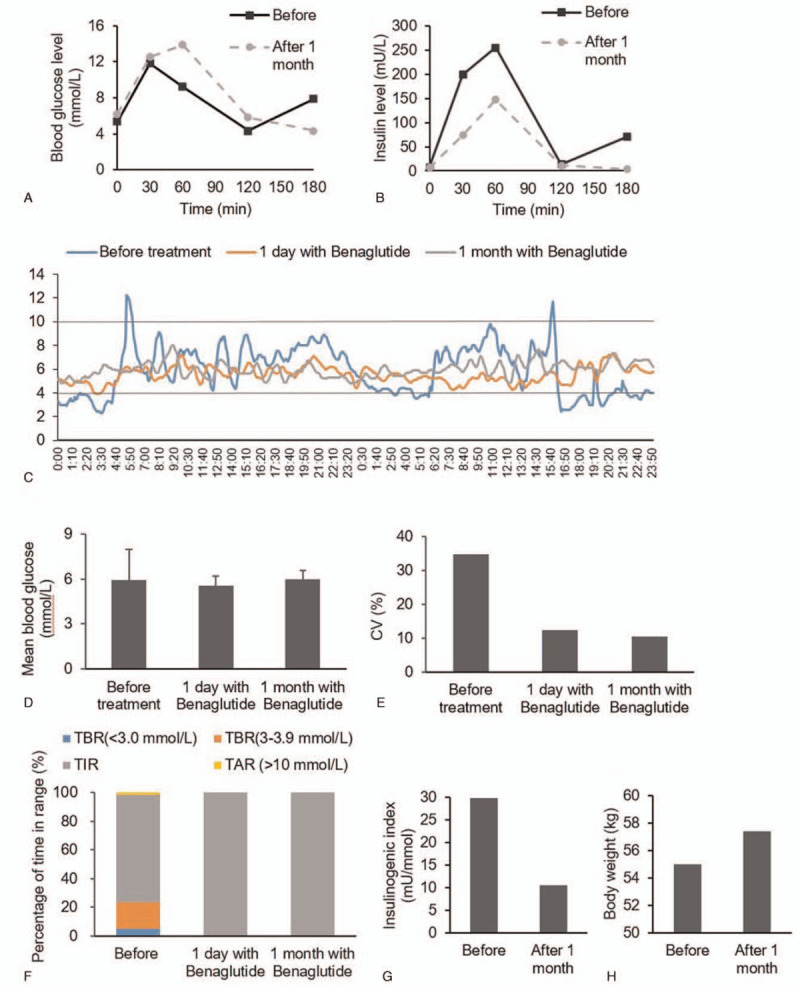
The glucose metabolism before and after beinaglutide treatment in the patient with late dumping syndrome. A. Blood glucose levels and B. insulin levels in OGTTs before and 1-month after beinaglutide treatment. C. Blood glucose profile in CGM at baseline, 1-day and 1-month after beinaglutide treatment. D. Mean blood glucose and standard deviation and E. coefficient of variation (CV) calculated with data from CGM. F. Percentage of time in range (TIR) of 3.9 to 10 mmol/L before and after beinaglutide treatment. G. Insulinogenic index = (insulin 30’ − insulin 0’)/(glucose 30’ − glucose 0’). H. The body weight before and after beinaglutide treatment factor-1.

On admission, the patient was 175 cm tall and weighed 55 kg, with a body mass index of 17.96 kg/m^2^. His blood pressure was 110/80 mmHg, his body temperature was 36.7 °C, and his pulse rate was 78 beats/min and regular. Physical examination showed no abnormal signs of note. His laboratory data on admission indicated no major abnormality (Table 1). Abdominal computed tomography indicated postoperative changes of the gastrointestinal tract. There was no evidence of a neuroendocrine tumor. The 75 g oral glucose tolerance test (OGTT) showed a fasting glucose level of 5.48 mmol/L and a fasting insulin level of 12.5 mU/mL. After glucose solution administration, the glucose level increased to a maximum of 11.83 mmol/L at 30 minutes, after which the glucose level declined slowly to 9.21 mmol/L at 60 minutes and 4.21 mg/dL at 120 minutes. The insulin peak of 255.9 mU/mL was observed at 90 minutes (Fig. 1A and B).

**Table 1:** Laboratory data on admission.

Fasting blood test		Normal range	Fasting blood test		Normal range
WBC (×10^9^/L)	3.38	3.5–9.5	Blood glucose, mmol/L	5.39	3.9–5.6
RBC (×10^12^/L)	4.15	4.3–5.8	Insulin, mU/L	7.7	2.3–11.6
Hemoglobin, g/L	135	130-175	CP, ng/mL	2.07	0.78–5.19
Platelet (×10^9^/L)	116	125-350	ACTH, pg/mL	16.5	0-46
ALT, U/L	50	9-50	Cortisol, μg/dL	11.35	2.9–17.3
AST, U/L	26	15-40	GH, ng/mL	0.187	<2.47
ALP, U/L	146	45-125	IGF-1, ng/mL	304.9	60-350
LDH, U/L	191	120-250			
BUN, mmol/L	4.64	2.86–8.20	Hormones when hypoglycemia		
Creatinine, μmol/L	54.0	5.3-123	Blood glucose, mmol/L	2.56	
Sodium, mmol/L	147.9	137-147	Insulin, mU/L	28.7	
Potassium, mmol/L	3.64	3.5–5.3	CP, ng/mL	6.23	
Calcium, mmol/L	2.16	2.11–2.52	ACTH, pg/mL	16.6	
TC, mmol/L	4.83	3.12–5.98	Cortisol, μg/dL	6.0	
Triglyceride, mmol/L	0.86	0.44–1.70	GH, ng/mL	8.18	
LDL-c, mmol/L	1.37	0.0–3.1	IGF-1, ng/mL	235.5	
HDL-c, mmol/L	2.21	1.0–1.9	Glucagon, pg/mL	152.71	
HbA1c, mmol/L	5.2	4.0–6.0			
TSH, mIU/L	1.355	0.35–4.94			
FT3, pmol/L	4.37	2.43–6.01			
FT4, pmol/L	11.34	9.0–19.0			

We also tested glucose responsive hormones when hypoglycemia occurred 1 hour after lunch on August 28, 2020. The patient had symptoms of palpitation and sweating with a blood glucose level of 2.56 mmol/L. The ratio of insulin/glucose was 0.62, and the response of cortisol, insulin-like growth factor-1 (IGF-1), and glucagon to hypoglycemia was not significant (Table 1).

The continuous glucose monitoring (CGM) on admission showed that 23.43% of all glucose values were in the hypoglycemic range of <3.9 mmol/L, and the time below the range (TBR) of <3.0 mmol/L was 5.03% (Fig. 1C and F). The time above the range of >10 mmol/L was 1.74 (%).

After the injection of beinaglutide 0.1 mg before breakfast and lunch instead of acarbose since Sep 1, 2020, the CGM showed that the time in range of 3.9 to 10 mmol/L (TIR) became 100%, and the standard deviation (SD) of 24-hour glucose and the coefficient of variation (CV) of glucose were significantly decreased although the 24-hour mean glucose did not change a lot (Fig. 1C–F).

The symptoms of paroxysmal palpitation, sweating, and dizziness have been in remission since beinaglutide treatment. OGTT and CGM were performed again after 1-month treatment of beinaglutide, and the insulin levels in the OGTT were decreased after beinaglutide treatment (Fig. 1B). The peak of glucose delayed to 60 minutes, while the peak of insulin remained at 60 minutes and decreased to 147.5 mU/L (Fig. 1B). We also tested glucagon levels in OGTT after 1 month, and the results showed that the fasting glucagon was 296.39 pg/mL and the lowest glucagon level was 170.67 at 180 minutes in the OGTT. After beinaglutide treatment for 1 month, the 24-hour mean blood glucose was 5.96 mmol/L and SD of blood glucose was 0.62 mmol/L. His CV was 10.47% and was even lower than that at the beginning of beinaglutide treatment (12.35%). The TIR of 3.9 to 10.0 mmol/L remained 100% (Fig. 1C–F). Moreover, the insulinogenic index was higher after 1-month treatment compared with baseline (Fig. 1G).

Moreover, there was no side effect, such as nausea, dizziness, or loss of appetite in this case. The patient had an unexpectedly improvement in his anxiety and gained 2.5 kg of weight after 1-month beinaglutide treatment (Fig. 1H).

The study was approved by the Institutional Ethical Committee of Nanjing First Hospital, Nanjing Medical University. Informed consent for publication of the case was obtained from the patient included in the case report.

## 3 Discussion

In this patient with dumping syndrome who had failed treatment with acarbose and diet modification, beinaglutide therapy showed good efficacy on glycemic variation, and the efficacy could persist for 1 month without any side effects.

Accelerated gastric emptying plays an important role in the pathogenesis of late dumping syndrome after gastric surgery, it gives rise to a larger and earlier increase in plasma glucose, insulin, GLP-1, and GIP concentrations, and thus to reactive hypoglycemia.^[3,8]^ GLP-1RAs can reduce gastric emptying, which leads to the reduction of hunger and food consumption in patients with type 2 diabetes or obesity.^[9]^ In patients with dumping syndrome, GLP-1RAs were also thought to have a therapeutic effect by inhibiting gastric emptying.^[5,10]^ In the present case, the peak of blood glucose was delayed, which may also attribute to the inhibition of gastric emptying, and the levels of insulin decreased significantly.

Liraglutide is a long-acting GLP-1RA, and it inhibited insulin secretion both before and after meals in a previously reported case with late dumping syndrome, and the TIR was only 84% after the treatment of 1.2 mg liraglutide per day,^[5]^ which was 100% in the present case. In another case report, Abrahamsson et al,^[7]^ treated 5 patients with postprandial hypoglycemia following gastric bypass surgery with liraglutide, and 2 of the patients experienced side effects including nausea and headache. The amino acid sequence of beinaglutide is the same as that of human GLP-1, with a half-life of about 11 minutes. Beinaglutide can be administered with meals flexibly, which also has an inhibitory effect on gastric empting, and can reduce insulin secretion within a short period of time after meals. Therefore, the treatment of beinaglutide may be more effective and have less side effects than the long-acting GLP-1RAs.

The benefits of beinaglutide in weight loss and glycaemic control have been confirmed in patients with type 2 diabetes.^[11]^ However, in this patient with a low body mass index (BMI) of 17.96 kg/m^2^, beinaglutide did not decrease his weight. On the contrary, he gained 2.5 kg after 1-month beinaglutide treatment because of the remission of anxiety symptoms and fear of hypoglycemia after eating.

The effects of beinaglutide on α and β cell function have not been well established since it was only approved by the China Food and Drug Administration for the treatment of T2DM in December 2016. The present case reported the first use of beinaglutide in a patient with dumping syndrome. We described the effect of beinaglutide on inhibiting hyperinsulinemia, which was opposite to the effect of GLP-1RAs on β-cell function in patients with type 2 diabetes.^[12]^ The patient showed a weak response of cortisol, IGF-1, and glucagon to hypoglycemia at baseline, which may attribute to recurrent hypoglycemia.^[13]^ After 1-month beinaglutide treatment, the glucagon levels were not suppressed. However, the glucagon level in OGTT at baseline was missing, which was a limitation. This case was also lack of data of GLP-1 and GIP levels before and after beinaglutide treatment. Previous studies found that inhibition of GLP-1 and GIP levels by octreotide can also improve dumping syndrome.^[14]^ Therefore, the detection of GLP-1 and GIP may further indicate the mechanism of beinaglutide in the treatment of dumping syndrome. Moreover, the long-term effects of beinaglutide on dumping syndrome need to be observed.

In conclusion, beinaglutide is an effective and safety treatment for late dumping syndrome. Our preliminary results suggest that the efficacy and mechanisms of beinaglutide in the treatment of dumping syndrome should be proved by further studies.

## Acknowledgments

The authors thank the members of Endocrinology department of Nanjing First Hospital for their support.

## Author contributions

**Conceptualization:** Jianhua Ma.

**Data curation:** Reng-Na Yan.

**Formal analysis:** Yun Hu.

**Investigation:** Bo Ding, Lu Yuan.

**Validation:** Jianhua Ma.

**Writing – original draft:** Yun Hu.

**Writing – review & editing:** Jianhua Ma.
